# Romantic Partners with Mismatched Relationship Satisfaction Showed Greater Interpersonal Neural Synchrony When Co-Viewing Emotive Videos: An Exploratory Pilot fNIRS Hyperscanning Study

**DOI:** 10.3390/neurosci6020055

**Published:** 2025-06-12

**Authors:** Wen Xiu Heng, Li Ying Ng, Zen Ziyi Goh, Gianluca Esposito, Atiqah Azhari

**Affiliations:** 1 Psychology Programme, School of Humanities and Behavioural Sciences, Singapore University of Social Sciences, Singapore 599494, Singapore; 2 Psychology Programme, School of Social Sciences, Nanyang Technological University, Singapore 639818, Singapore; 3 Department of Psychology and Cognitive Science, University of Trento, 38068 Trento, Italy

**Keywords:** emotional attunement, interpersonal neural synchrony, relationship satisfaction, functional near-infrared spectroscopy

## Abstract

Emotional attunement, or emotional co-regulation in a relationship, can manifest as interpersonal neural synchrony, where partners exhibit similar anti-phase or phase-shifted brain activity. In adult romantic relationships, emotional attunement may differ according to relationship satisfaction. No study has examined how relationship satisfaction difference influences interpersonal neural synchrony. This exploratory pilot study on 17 couples (unmarried Chinese undergraduate couples in a Southeast Asian university) investigated whether relationship satisfaction difference influenced interpersonal neural synchrony during a shared emotive experience. Each couple wore an fNIRS cap to measure brain activity in their prefrontal cortex (PFC) while co-viewing seven videos intended to evoke positive, negative or neutral emotions. We found preliminary evidence that relationship satisfaction difference modulated interpersonal neural synchrony in the right ventral PFC regions, including the right ventromedial PFC (involved in the encoding of emotional values to stimuli and emotional regulation), right ventrolateral PFC (involved in voluntary emotional regulation) and the right orbitofrontal cortex (involved in processing of emotional experiences and regulation of emotions). This suggested that couples with mismatched relationship satisfaction displayed greater interpersonal neural synchrony, possibly due to mutual social cognitive processes when viewing emotive videos together. Further studies can replicate the findings with larger, diverse samples.

## 1. Introduction

Emotional attunement refers to the two-way process of regulating one’s emotion in response to another party’s behavioural changes (e.g., facial expression, body language, or tone of voice) in an interpersonal relationship, through emotional arousal or dampening, so that both partners in the relationship experience emotional stability [1]. It is a form of co-processing emotional events, particularly negative ones, responding to the other party (e.g., mirroring), and then moving on together to a more positive emotional state [2]. It indicates the level of emotional responsiveness and connectedness between two persons in a relationship, where partners are turning towards each other emotionally [2]. Broadly, emotional attunement is important because co-regulation to each other’s positive affect facilitates bonding and empathy between partners [3].

Such emotion regulation can involve real-time regulation of one’s emotions in response to the other party’s emotions, known as emotional attunement, or more enduring adjustment of one’s mood to the other party’s, known as coupling [4]. Attunement occurs during shared experiences such as the dynamic process of a social interaction and can manifest externally as mimicry or mirroring of the other party’s facial expressions and behaviours [5], which has been shown to improve social interactions [6].

### 1.1. Interpersonal Neural Synchrony as a Measure of Emotional Attunement

Recent neurophysiological studies have shown that beyond external behavioural and physiological responses, emotional attunement can be detected in interpersonal neural synchrony between two persons’ brain activity, such as between parent and child [7,8], between co-parents [9] and between partners on a collaborative task [10]. Prochazkova and Kret further proposed that interpersonal neural synchrony activates mirroring of physiological responses, which in turn activates emotional attunement [11]. Interpersonal neural synchrony refers to the temporal alignment between two time-series sequences and includes both anti-phase and phase-shifted brain activity [12].

There are also studies on emotional regulation processes in romantic relationships, such as between married or dating couples [13,14], another key demographic in this research field on interpersonal neural synchrony. Romantic relationships have similar features to mother–child relationships, such as being highly interdependent in nature [15] and having strong multi-dimensional connections [1], including emotional contagion [4], yet are distinct in that these relationships are between two adults with romantic interest towards each other as defined in this study. Butner and colleagues [4] similarly found that their sample of couples showed covariation in both positive and negative affect, as well coupling for positive affect. In addition, covariation was greater in couples who reported spending more time together, possibly due to greater levels of emotional contagion. Azhari and colleagues [16] employed fNIRS to investigate the interpersonal neural synchrony of 24 co-parenting spousal dyads. This study showed that the presence of a co-parenting spouse in the same room was associated with greater synchrony of brain activity in the PFC towards aural stimuli, compared to the presence of a co-parenting spouse in a separate room or the presence of a stranger in the same room. These studies suggested physiological and psychological synchrony as a proxy for emotional attunement in couples in romantic relationships.

Specifically, the PFC includes the ventromedial PFC, the ventrolateral PFC and the orbitofrontal cortex, which are regions broadly associated with emotion processing and regulation. Brain imaging studies have found that the ventromedial PFC shows higher activity levels in response to emotion-provoking images, suggesting its involvement in encoding of emotions in response to external stimuli and regulation of emotions [17]. The ventrolateral PFC has been suggested to be a critical brain region involved in voluntary emotion regulation [18], with specifically the right ventrolateral PFC associated with regulating emotions associated with social pain [19]. The orbitofrontal cortex also contributes to emotion regulation, particularly through the reappraisal of negative emotion-provoking stimuli [20].

### 1.2. Association Between Emotional Attunement and Relationship Satisfaction

The finding that interpersonal neural synchrony was greater in spouses than in strangers, suggested that certain aspects of a romantic relationship may moderate the link between relationships and emotional attunement. Watson and colleagues [21] suggested that similarity of attitudes and backgrounds strongly predicted relationship outcomes such as relationship satisfaction. Extending from this finding, similarity of attitudes towards the relationship itself may be associated with relationship satisfaction. Li et al. found that interpersonal neural synchrony as a result of emotional attunement strongly predicted levels of relationship satisfaction in married couples [22].

Relationship satisfaction refers to one’s feelings, thoughts and behaviours in a relationship [23], or simply one’s overall assessment of the relationship [24]. Past studies looked at the link between the level of relationship satisfaction with other relationship outcomes, including emotional attunement [22]. Low relationship satisfaction has been shown to be associated with reduced commitment towards one’s partner, increased likelihood of looking for other relationships [25] and increased emotional distress [26]. On the other hand, high relationship satisfaction in romantic couples was associated with greater regulation of one’s partner’s emotions to help the partner to feel better [27]. However, there remains a gap in the field in understanding if differences in relationship satisfaction between partners would predict interpersonal neural synchrony, potentially due to shared social attention [28].

### 1.3. Emotion Attunement and Emotional Valence

Emotional attunement has also been found to be positively associated with physiological synchrony, such as partners exhibiting significant coordination in their physiological responses (e.g., perspiration rate and heart rate) [29,30,31]. These studies have confirmed the presence of notable physiological synchrony during couple interactions, underscoring the depth of psychophysiological interconnections within romantic partnerships. Interestingly, these investigations have revealed variations in the levels of physiological synchrony depending on the emotional context of the interactions. Lin and colleagues [32] argue that in interpersonally close dyads, when partners’ emotions are synchronised, their physiology would also be more synchronised, and their emotional valence would vary in a similar direction when presented with emotionally provoking stimuli (regardless of positive or negative emotions elicited).

### 1.4. Aim and Hypothesis of Present Study

In our current study, we utilised functional near-infrared spectroscopy (fNIRS) as a primary tool to investigate brain activity in participants during shared activities. The choice of fNIRS is informed by its advantages for examining interpersonal neural synchrony in romantic relationships. This non-invasive technique is well-suited for naturalistic, interactive settings due to its relative tolerance to movements, as opposed to the more restrictive environments required by fMRI (functional magnetic resonance imaging), which is crucial for studying real-time interpersonal dynamics, where natural movement is integral [33].

Broadly, fNIRS provides a balance between spatial and temporal resolution, which is essential for capturing the dynamic neural processes underlying emotional attunement and synchrony. While it does not offer the spatial resolution of fMRI, fNIRS provides sufficient resolution to identify activity in key cortical areas involved in social and emotional processing, such as the PFC [34]. Furthermore, its superior temporal resolution allows for the detection of rapid changes in cortical haemodynamics associated with fast-paced interpersonal interactions [34].

Recent hyperscanning studies have demonstrated the potential of fNIRS in revealing synchronised brain activity in the prefrontal regions of romantic partners during cooperative tasks or shared emotional moments [35,36,37,38]. These studies affirm that such synchronisation signifies a deeper alignment in emotional and cognitive states, crucial for successful interpersonal engagement [39,40].

Our research aimed to contribute to this evolving field by examining neural synchrony in romantic relationships within the cultural context of Singaporean Chinese non-married couples, specifically to uncover how differences in romantic partners’ relationship satisfaction might correlate with the neural synchrony patterns of couples. This study investigated whether the matching of relationship satisfaction within a romantic relationship influenced emotional attunement in a couple, as marked by the extent of synchrony between a couple’s brain activity while performing a shared activity. Specifically, this study looked at the interpersonal neural synchrony of a romantically linked couple when viewing video stimuli intended to evoke either positive or negative emotions. The hypotheses were as follows:

(a) There would be differences in the interpersonal neural synchrony of the brain activity of the two partners in a romantic relationship during the shared co-viewing activity across the positive emotion condition, the negative emotion condition and the baseline condition.
(b) The smaller the difference in the romantic partners’ relationship satisfaction levels (regardless of the extent of relationship satisfaction levels themselves), the greater the interpersonal neural synchrony in the brain activity of the two partners in a romantic relationship during the shared co-viewing activity.

## 2. Materials and Methods

### 2.1. Participants

The sample comprised 17 Chinese, heterosexual (i.e., male–female), non-married couples (undergraduate students) recruited from a Singapore university. The age for one of the couples was not captured. The mean ages for the remaining 16 couples were 22.8 years (for males) and 21.6 years (for females) (range: 18–24 years). The mean duration of the couples’ romantic relationship duration was 65.6 weeks (range: 4–158 weeks; standard deviation [SD]: 55.3 weeks). All participants provided informed consent prior to their participation. They were offered reimbursement of USD 15 or partial course credit for their participation in the study. Data from two couples were excluded due to technical errors, so only 15 couples’ data was included in the analysis. Data for this study is available on at https://osf.io/fe87j/?view_only=7cc90468ea714e859d218bcb62a69d50 (accessed on 28 May 2025).

### 2.2. Study Design

**Figure 1:**
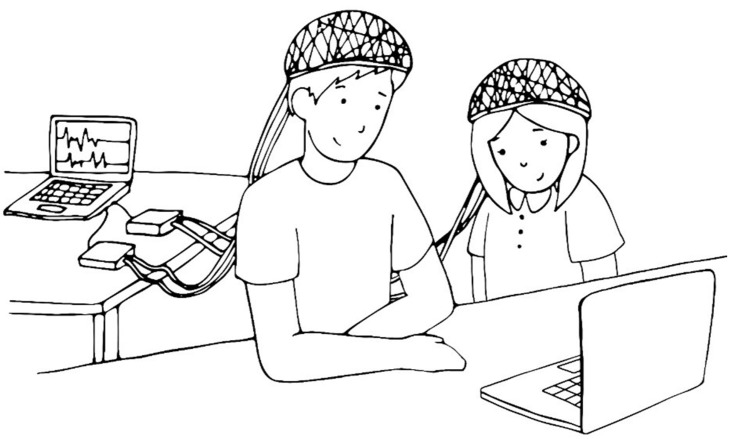
Illustration of the set-up in the lab.

Ethics approval was obtained from the Division of Psychology’s ethics committee and the study was conducted in accordance with the Declaration of Helsinki. In the first part of the study, all selected participants were asked to fill in the 7-item Relationship Assessment Scale (see Table 1 for the questions; Cronbach’s alpha = 0.91 [41]) [42] online individually. Following which, a lab session was conducted for each of the individual couples. During the lab session, the couple was seated adjacent to each other in a closed room. A 13-inch laptop was set up on the desk in front of the seated couple. Each participant was fitted with an NIRS cap attached to a NIRSport device by an experimenter. Each participant was also instructed to put on separate earphones that were linked to the same output with an earphone splitter, and the experimenter checked that they were able to hear the output clearly. Each participant was asked to keep their gaze on the video stimulus on the laptop monitor. Figure 1 shows the set-up of the lab. The couple was told not to interact with each other in any way while watching the video stimulus, and the experimenter kept a watch on the couple during the experiment to ensure this condition was met. The experimenter then turned off the lights and left the room. At the end of the experiment, the participants were debriefed by the experimenter before leaving the lab.

### 2.3. Video Stimuli

Each couple was randomly assigned to one of three random sequences of seven video stimuli. See Table 2 for the three sequences. Five couples were assigned to Sequence A; five to Sequence B; and four to Sequence C.

**Figure 2:**
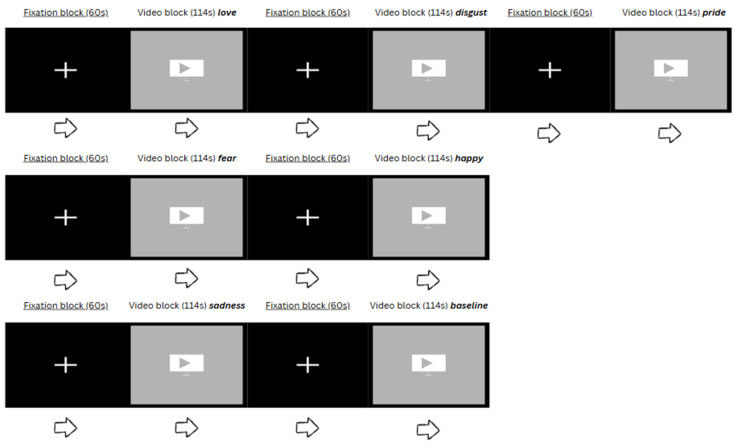
Example of a random sequence of seven video stimuli.

For each sequence, there was a fixation block lasting 60 s, followed by a video stimulus lasting 114 s, and this pattern was repeated till all seven video stimuli were shown. The total duration was 20 min 18 s. Figure 2 below illustrates one of the three random sequences. The fixation block was intended as a break, so that participants could revert to baseline levels of emotions.

The video stimuli were video clips taken from the YouTube platform (https://www.youtube.com). The video stimuli were intended to evoke specific emotions in viewers—in a pre-test, the evoked emotions were assigned by two independent raters and their assignments concurred (Cohen’s kappa > 0.08). Of the seven video stimuli, three were intended to evoke positive emotions (i.e., happiness, love or pride); three to evoke negative emotions (i.e., sadness, fear or disgust); and one was a neutral stimulus that was not intended to evoke any emotions. See Table 3 for the list of emotions and corresponding video stimuli.

### 2.4. Functional Near-Infrared Spectroscopy (fNIRS) Data Preprocessing and Analysis

**Figure 3:**
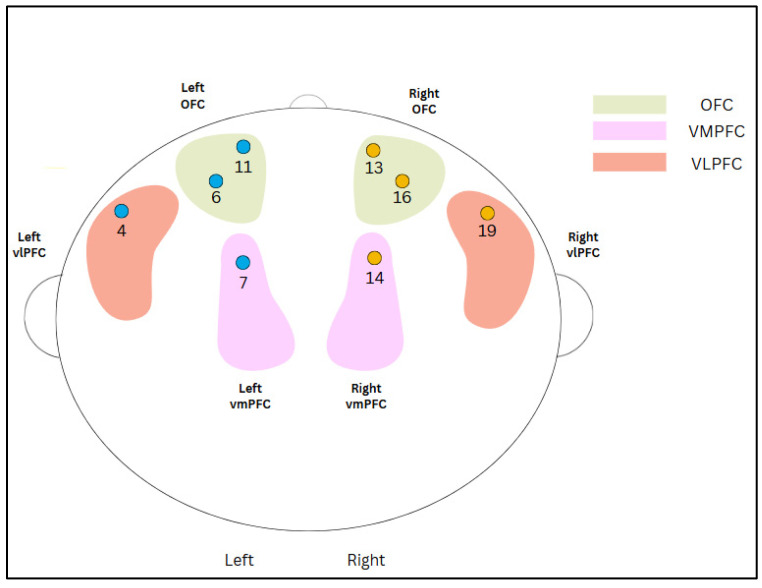
Illustration of the 8 channels used and their corresponding targeted brain regions. Channels 4 and 19 correspond to the left and right VLPFC, respectively. Channels 7 and 14 correspond to the left and right VMPFC, respectively. Channels 6 and 11 correspond to the left OFC, and 13 and 16 correspond to the right OFC.

Neural activity in the PFC was captured via the fNIRS neuroimaging system. Each NIRScap had 8 LED sources and 7 detectors to pick up signals optically in a non-invasive manner. The LED sources emitted light with wavelengths of 760 nm and 850 nm; each detector scanned at the rate of 7.81 Hz. The distance between detectors was 3 cm. The NIRSlab software (NIRS v.205 software) used a standard 8 × 7 source-detector montage to set up a 20-channel recording system of the PFC [43]. As this study focuses on the ventral frontal and orbitofrontal cortices (i.e., ventromedial PFC, ventrolateral PFC and orbitofrontal cortex—involved in emotion processing and regulation), only data from 8 channels were included in the analysis (see Figure 3). As fNIRS measures the level of oxygen in blood in specific brain parts, it derived the concentrations of oxygenated haemoglobin and reduced haemoglobin to illustrate the differences in brain activity in different parts of the brain.

Pre-processing and analysis of the data collected was conducted using the NIRSlab software. For pre-processing, data with gain > 8 and CV > 7.5 was interpreted as background noise and filtered out from the dataset. In addition, the time marker for the start of the video stimuli was added to the dataset. Based on these time markers, outlying data such as discontinuities and spike artefacts were removed. The data was then filtered through a band-pass of 0.01–0.2 Hz, to remove noise such as low signals and baseline shift variations. The data was then converted to concentrations of oxygenated haemoglobin and reduced haemoglobin based on the Beer–Lambert Law, deriving a haemodynamic response function. As a final check, two independent coders manually checked the dataset for potential artifacts.

Following Azhari et al. [43], Dynamic Time Warping, using the R *dtw* package, was then employed to quantify the pre-processed time-series data to generate normalized distance indexes in each channel within each couple. This algorithm transformed the data such that similarly shaped data (even if out-of-phase) that occurred at the same time were matched, resulting in arrangements of sequences that optimised the match between the couple. In the R *dtw* package, the normalized distance index was computed by dividing the total dynamic time-warping (DTW) distance by the combined length of the two time series (i.e., the sum of the lengths of both sequences). This standardisation also accounts for differences in sequence duration and enables meaningful, scale-independent comparisons. The method is consistent with the implementation used in previous studies, such as that by Azhari et al. [43], where the normalized DTW distance facilitates robust similarity assessment across biological time-series data. The normalized distance index was computed for each channel, for which the greater the distance of the index, the lower the extent of interpersonal neural synchrony between the couple. The channels were then aggregated into two regions of interest (ROIs) of the PFC: the frontal right and the frontal left (i.e., the ventromedial PFC, ventrolateral PFC and orbitofrontal cortex associated with emotion processing and regulation), according to the methods in Azhari et al. [43].

### 2.5. Analytical Plan

Given the small sample size of 15 couples in this exploratory study, we focused on the normalized distance indexes in the frontal left and right clusters only, which includes the left and right (respectively) ventromedial PFC, ventrolateral PFC and orbitofrontal cortex. Similarly, due to the small sample size, the distance indexes were averaged across positive emotions (i.e., happiness, love and pride) and negative emotions (i.e., sadness, fear and disgust).

Kruskal–Wallis analysis was conducted to investigate if there was any difference in normalized distance indexes across the positive, negative and baseline conditions. Multiple linear regression analyses were conducted to examine if romantic partners’ relationship satisfaction score difference and type of emotion evoked by the videos (i.e., only positive and negative conditions were included) predicted the distance index for each brain cluster. Pearson’s correlation analysis was conducted to examine the relationship between the relationship satisfaction score difference and the normalized distance indexes for the frontal right cluster.

## 3. Results

The mean satisfaction score of male participants was 4.3 out of 7 (SD: 0.5), while that of female participants was 4.4 (SD: 0.5). The 15 participating couples showed an average of 0.58 difference (SD: 0.47) in romantic partners’ relationship satisfaction scores. The male participants scored an average of 4.30 in their relationship satisfaction, and the female participants similarly scored an average of 4.45 in their relationship satisfaction.

### 3.1. Difference in Interpersonal Neural Synchrony Across Conditions

The results of the Kruskal–Wallis analysis showed a potential difference in the distance index across the positive, negative and baseline conditions, though the difference was not statistically significant in this study [*H*(2, *n* = 15) = 5.26, *p* = 0.07]. However, there was a significant difference in the distance index across the emotion-evoking condition (i.e., combining positive and negative conditions) and baseline condition [*H*(1, *n* = 15) = 3.88, *p* = 0.04].

### 3.2. Relationship Between Relationship Satisfaction and Interpersonal Neural Synchrony

**Figure 4:**
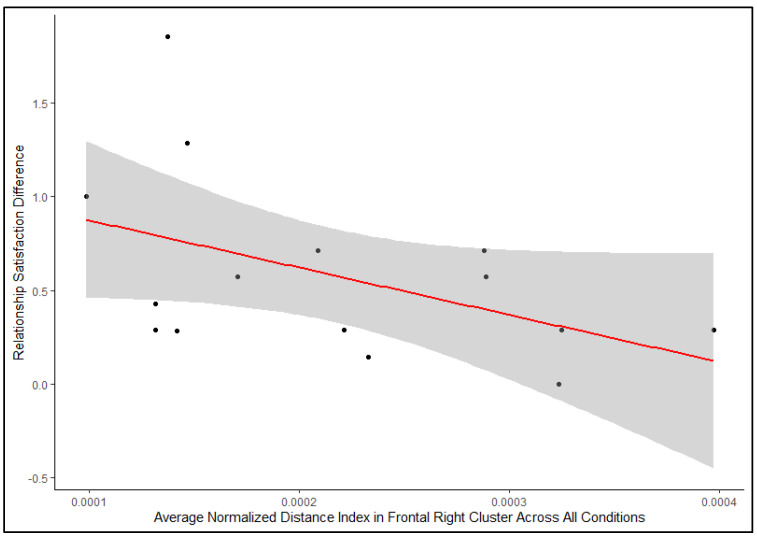
Scatterplot of the negative linear relationship between relationship satisfaction difference between partners and averaged normalized distance index of the frontal right brain cluster in couples [r = −0.47, *p* = 0.079].

The results of the regression models showed that couples’ relationship satisfaction score difference negatively predicted the distance index in the frontal right cluster of the PFC [t(27) = −2.31, *p* = 0.028] (Table 4a), but neither the relationship satisfaction score difference nor the type of emotions evoked predicted the distance index in the frontal left cluster of the PFC (Table 4b). The Pearson’s correlation analysis (Figure 4) further showed that a negative relationship between the relationship satisfaction score difference negatively predicted the normalized distance index in the frontal right cluster, though this relationship was not statistically significant [r = −0.47, *p* = 0.079]. This means that the greater the difference in self-reported relationship satisfaction between partners, the lower the distance index and, correspondingly, the greater the interpersonal neural synchrony in the frontal right brain cluster. The frontal right cluster includes the right ventromedial PFC, right ventrolateral PFC and the right orbitofrontal cortex (see supplementary analyses in Table S1).

## 4. Discussion

This study investigated if there was a difference in interpersonal neural synchrony between romantically linked partners when viewing videos intended to evoke either positive or negative emotions or a neutral video, and if the difference in self-reported relationship satisfaction was connected to their interpersonal neural synchrony when viewing videos intended to evoke either positive or negative emotions. The hypotheses were that (1) there would be a difference in interpersonal neural synchrony across the three conditions, and (2) the smaller the difference in the couple’s relationship satisfaction levels, the greater the interpersonal neural synchrony. The first hypothesis was partially supported, in that there was a significant difference between the emotion-evoking conditions and the baseline condition, while our findings were contrary to the second hypothesis. This study unveiled a potential link between relationship satisfaction difference and interpersonal neural synchrony when romantic partners undergo shared experiences. Specifically, interpersonal neural synchronisation was observed in the frontal right brain cluster, which includes the right ventromedial PFC, right ventrolateral PFC and the right orbitofrontal cortex, which are involved in emotion processing and regulation [17,18,19,20], more so in couples with greater differences in their relationship satisfaction.

The brain areas involved are also recognised for their role in social decision-making [44] and social behaviours underpinning cooperation [45]. Indeed, the frontal cluster of the PFC is pivotal in interpreting social cues, predicting others’ emotional reactions, and adjusting one’s own behaviour accordingly to navigate complex interpersonal situations. Thus, a potential interpretation for our finding is that, in mismatched couples, where one partner is more satisfied than the other, both individuals may subconsciously recognise a lack of emotional alignment and exert greater cognitive effort in socially oriented processes governed by the frontal cluster of the PFC to calibrate their personal emotions with those of their partners [45,46] and regulate their emotions to each other to achieve emotional stability [1,2]. For example, the more satisfied partner might anticipate how their reactions could affect the less satisfied one, while the less satisfied partner might monitor the other’s emotions to manage tension or improve connection. This mutual but asymmetrical engagement in emotional and social strategising could lead to heightened neural synchrony in the right frontal regions. By contrast, couples with equally high satisfaction do not need to exert as much effort to plan or regulate their interactions because their emotional understanding is more automatic. Meanwhile, equally low-satisfaction couples may disengage from the emotional or social needs of the relationship altogether, showing less brain activity in regions involved in coordination or mutual adjustment. Thus, the increased synchrony in mismatched couples likely reflects an intensified use of the frontal right cluster for real-time emotional coordination during social activities.

This association between greater brain synchrony in the frontal regions of the PFC and asymmetrical relationship quality has also been observed in a recent study on parent–child dyads, where parents who reported greater parenting stress displayed heightened synchrony with their child in the frontal regions of the PFC, but lower synchrony in the posterior regions of the PFC [47]. In that study, the authors suggested that the region-specific differences in synchrony patterns may have emerged due to the different functions of the frontal and posterior areas of the PFC. While the posterior cluster of the PFC is more involved in mentalisation and attunement, the frontal areas oversee social decision-making and task planning. Heightened synchrony in these frontal areas may serve as a compensatory mechanism to actively coordinate social decision-making to achieve greater dyadic regulation. Our results suggest similar synchrony mechanisms in romantic dyads with mismatched relationship satisfaction.

In extrapolating the implications of our findings, a shared neurological framework specifically within a romantic couple could facilitate mutual understanding [2,3] through collaborative navigation of shared life experiences, including shared experiences that do not involve overt social interaction [48] or physical touch [49]. The synchronised neural patterns discerned in our research might be emblematic of the intertwined emotional and cognitive realities that partners continually strive to forge, driven by emotional attunement, regulation and collaboration. These elements could be foundational to thriving romantic bonds, given that these actions are associated with nurturing successful interpersonal connections [4].

### Limitations and Future Directions

Our study, while offering noteworthy findings, comes with its set of limitations. A significant constraint is our sample size, predominantly drawn from a university demographic in Singapore and limited to heterosexual, non-married relationships with a wide range of relationship lengths. Given the small sample size, the preliminary results should be interpreted cautiously. There could be potential differences in high versus low relationship satisfaction, despite similarity (or lack thereof) within the couple [22], and in the relationship quality and emotional attunement among couples who had been dating for different lengths of time, in different developmental stages (e.g., young adults versus mature adults), and in different relationship stages (e.g., dating versus married) or type (exclusive versus open relationships). There could also be differences between non- or passive social interactions (as per this study) and active social interactions such as those discussed in [42] which can be further explored. Replication of the study with larger and more diverse samples could also explore the non-significant differences across emotion conditions, and investigate each of the individual emotions evoked through the videos and whether participants did experience the intended emotion, as the emotions within each category (e.g., love versus happiness) could have resulted in different brain activity changes. Checking the emotions evoked by each video could also help to detect possible spillover of emotions from an earlier video to a later one.

Future research should consider more diverse and bigger samples, accounting for different cultures and relationship dynamics. Future studies could also include a power analysis to determine a suitable sample size and include further analyses such as interaction terms in the regression models. In addition, future studies could consider exploring other brain regions that may be involved in emotion regulation.

## 5. Conclusions

Our research found connections between relationship satisfaction differences and interpersonal neural synchrony during shared experiences in romantic partners, primarily in the frontal right brain cluster. However, the study’s confines, especially its small sample size and homogeneous sample profile, emphasises the need for extended research. A deeper exploration encompassing diverse cultural and relational spectrums will be instrumental in furnishing a more holistic picture of emotional attunement’s intricacies in romantic relationships.

## Figures and Tables

**Table 1:** Relationship Assessment Scale (Hendricks, 1998) [42].

7-Item Relationship Satisfaction Questionnaire, Using 7-Point Likert Scale
How well does your partner meet your needs?
In general, how satisfied are you with your relationship?
How good is your relationship compared to most?
How often do you wish you hadn’t gotten into this relationship?
To what extent has your relationship met your original expectations?
How much do you love your partner?
How many problems are there in your relationship?

**Table 2:** Order of video stimuli for each sequence.

Sequence	Order of Video Clips
A	Love, Disgust, Pride, Fear, Happy, Sadness, Baseline
B	Sadness, Love, Baseline, Pride, Disgust, Fear, Happy
C	Baseline, Sadness, Disgust, Happy, Fear, Pride, Love

**Table 3:** Video description for each video stimulus.

Emotion	Video Description
Happiness	A self-filmed video of a Korean child giving innocent answers to her parents’ questions on safety issues—it was intended to showcase the innocence of a young child
Love	A chewing-gum advertisement depicting how a couple’s romantic relationship evolved and strengthened through a period
Pride	A video of the 100 m butterfly men’s swimming event at the 2016 Summer Olympics, where a swimmer clinched Singapore’s first Gold medal–a significant event for Singaporean viewers
Sadness	A video which started with a girl happily celebrating her birthday, but progressed to war times in her hometown, where she gradually lost the things around her and lost her smile
Fear	A video of a woman fearing noises and flickering lights while alone at home in the dark, ending with an unexpected creature in her room at the end of the video
Disgust	A short documentary on the unexpected contents of McNuggets, including less-preferred parts of a chicken (e.g., cartilage and fats) and non-food (e.g., wire bits)
Neutral	A video of calm and repetitive ocean currents

**Table 4:** (**a**). Multiple linear regression model for normalized distance index–frontal right cluster. (**b**). Multiple linear regression model for normalized distance index–frontal left cluster.

(a)				
Variable	Estimate	Standard Error	t-Value	*p*-Value
(Intercept)	0.0003	0.00003	8.49	<0.001
Romantic partners’ relationship satisfaction score difference	−0.00009	0.00004	−2.31	0.028 *
Type of emotion (positive)	−0.00003	0.00004	−0.81	0.42
**(b)**				
**Variable**	**Estimate**	**Standard error**	**t-value**	***p*-value**
(Intercept)	0.0003	0.00003	9.19	<0.001
Romantic partners’ relationship satisfaction score difference	−0.00005	0.00003	−1.63	0.11
Type of emotion (positive)	−0.00006	0.00003	−1.75	0.09

* Denotes statistical significance at <0.05.

## Data Availability

Data for this study is available at https://osf.io/fe87j/?view_only=7cc90468ea714e859d218bcb62a69d50 (accessed on 28 May 2025).

## Supplementary Materials

The following supporting information can be downloaded at https://www.mdpi.com/article/10.3390/neurosci6020055/s1, Table S1: Test for interaction between relationship satisfaction difference and emotion evoked. Table S2: (a) Multiple linear regression model for interpersonal neural synchrony—frontal right cluster. (b) Multiple linear regression model for interpersonal neural synchrony—frontal left cluster.
