# Supplementary material for: Romantic Partners with Mismatched Relationship Satisfaction Showed Greater Interpersonal Neural Synchrony When Co-Viewing Emotive Videos: An Exploratory Pilot fNIRS Hyperscanning Study
Source: NeuroSci. 2025 Jun 12;6(2):55. doi: 10.3390/neurosci6020055 (PMC12196001; doi:10.3390/neurosci6020055)
Supplement: Supplementary file 1 [file neurosci-06-00055-s001.zip › neurosci-3630111-supplementary.pdf]

Supplementary Materials

Table S1. Test for interaction between relationship satisfaction difference and emotion evoked.

Table S1a

Multiple linear regression model for interpersonal neural synchrony – frontal right cluster

Variable	Estimate	Standard error	t-value	p-value
(Intercept)	0.0003	0.00004	7.16	<.001
Romantic partners' relationship satisfaction score difference	-0.0001	0.00005	-1.84	.08
Type of emotion (positive)	-0.00004	0.00006	-0.76	0.46
Interaction between relationship satisfaction score difference and emotion	0.00002	0.00008	0.32	0.75

** Denotes statistical significance at <.05*

Table S1b

Multiple linear regression model for interpersonal neural synchrony – frontal left cluster

Variable	Estimate	Standard error	t-value	p-value
(Intercept)	0.0003	0.00003	8.60	<.001

Romantic partners' relationship satisfaction score difference	-0.0001	0.00005	-2.18	.039*
Type of emotion (positive)	-0.0001	0.00005	-2.23	.035*
Interaction between relationship satisfaction score difference and emotion	0.00009	0.00007	1.42	0.17

** Denotes statistical significance at <.05*

(1) Test for sequence effect

Table S2a

Multiple linear regression model for interpersonal neural synchrony – frontal right cluster

Variable	Estimate	Standard error	t-value	p-value
(Intercept)	0.0003	0.00005	4.73	<.001
Romantic partners' relationship satisfaction score difference	-0.00006	0.00005	-1.26	.22
Type of emotion (positive)	-0.00003	0.00003	-0.83	0.41
Condition_B (covariate)	-0.00003	0.00004	-0.64	0.53
Condition_C (covariate)	0.00005	0.00005	1.02	.32

** Denotes statistical significance at <.05*

Table S2b

Multiple linear regression model for interpersonal neural synchrony – frontal left cluster

Variable	Estimate	Standard error	t-value	p-value
(Intercept)	0.0002	0.00005	5.36	<.001
Romantic partners' relationship satisfaction score difference	-0.00003	0.00004	-0.64	.53
Type of emotion (positive)	-0.00006	0.00003	-1.86	0.07
Condition_B (covariate)	-0.00004	0.00004	-0.98	0.34
Condition_C (covariate)	0.00005	0.00005	1.19	.25

* *Denotes statistical significance at <.05*
